# Comparative Study of NGS Platform Ion Torrent Personal Genome Machine and Therascreen Rotor-Gene Q for the Detection of Somatic Variants in Cancer

**DOI:** 10.3390/ht9010004

**Published:** 2020-02-11

**Authors:** Angela Lombardi, Margherita Russo, Amalia Luce, Floriana Morgillo, Virginia Tirino, Gabriella Misso, Erika Martinelli, Teresa Troiani, Vincenzo Desiderio, Gianpaolo Papaccio, Francesco Iovino, Giuseppe Argenziano, Elvira Moscarella, Pasquale Sperlongano, Gennaro Galizia, Raffaele Addeo, Alois Necas, Andrea Necasova, Fortunato Ciardiello, Andrea Ronchi, Michele Caraglia, Anna Grimaldi

**Affiliations:** 1 Department of Precision Medicine, University of Campania “L. Vanvitelli”, 80138 Naples, Italy; marghe.russo88@libero.it (M.R.); amalia.luce@unicampania.it (A.L.); floriana.morgillo@unicampania.it (F.M.); gabriella.misso@unicampania.it (G.M.); erika.martinelli@unicampania.it (E.M.); teresa.troiani@unicampania.it (T.T.); fortunato.ciardiello@unicampania.it (F.C.); michele.caraglia@unicampania.it (M.C.); anna.grimaldi@unicampania.it (A.G.); 2 Section of Histology, Department of Experimental Medicine, University of Campania “Luigi Vanvitelli”, 80138 Naples, Italy; virginia.tirino@unicampania.it (V.T.); vincenzo.desiderio@unicampania.it (V.D.); gianpaolo.papaccio@unicampania.it (G.P.); 3 Department of Cardiothoracic Surgery, University of Campania “L. Vanvitelli”, 80138 Naples, Italy; francesco.iovino@unicampania.it; 4 Dermatology Unit, Department of Mental Health and Physics and Preventive Medicine, University of Campania Luigi Vanvitelli Naples, 80100 Napoli, Italy; giuseppe.argenziano@unicampania.it (G.A.); elvira.moscarella@unicampania.it (E.M.); 5 Division of Gastrointestinal Tract Surgical Oncology, Department of Translational Medical Sciences, University of Campania ‘L. Vanvitelli’, 80100 Naples, Italy; pasquale.sperlongano@unicampania.it (P.S.); gennaro.galizia@unicampania.it (G.G.); 6 Division of Medical Oncology, ‘San Giovanni Di Dio Hospital’, ASL NA2NORD, 80100 Naples, Italy; raffaeleaddeo19@gmail.com; 7 CEITEC—Central European Institute of Technology, University of Veterinary and Pharmaceutical Sciences Brno, 602 00 Brno, Czech Republic; necasa@vfu.cz (A.N.); necasovaa@vfu.cz (A.N.); 8 Division of Pathology, Department of Mental Health and Physics and Preventive Medicine, University of Campania Luigi Vanvitelli, 80100 Naples, Italy; andrea.ronchi@unicampania.it; 9 Biogem Scarl, Institute of Genetic Research, Laboratory of Precision and Molecular Oncology, Contrada Camporeale, 83031 Ariano Irpino (AV), Italy

**Keywords:** ion torrent personal genome machine (PGM), therascreen rotor gene Q, non-small cell lung cancer (NSCLC), metastatic colorectal cancer (mCRC), melanoma

## Abstract

Molecular profiling of a tumor allows the opportunity to design specific therapies which are able to interact only with cancer cells characterized by the accumulation of several genomic aberrations. This study investigates the usefulness of next-generation sequencing (NGS) and mutation-specific analysis methods for the detection of target genes for current therapies in non-small-cell lung cancer (NSCLC), metastatic colorectal cancer (mCRC), and melanoma patients. We focused our attention on EGFR, BRAF, KRAS, and BRAF genes for NSCLC, melanoma, and mCRC samples, respectively. Our study demonstrated that in about 2% of analyzed cases, the two techniques did not show the same or overlapping results. Two patients affected by mCRC resulted in wild-type (WT) for BRAF and two cases with NSCLC were WT for EGFR according to PGM analysis. In contrast, these samples were mutated for the evaluated genes using the therascreen test on Rotor-Gene Q. In conclusion, our experience suggests that it would be appropriate to confirm the WT status of the genes of interest with a more sensitive analysis method to avoid the presence of a small neoplastic clone and drive the clinician to correct patient monitoring.

## 1. Introduction

Carcinogenesis is a multiphase process that drives the progressive transformation of a normal cell into a tumor cell. Decades of research on cancer have proven that it is caused by mutations accumulated in various genes that control tumor initiation and progression [1,2]. 

The detection of somatic mutations in primary tumors represents a critical point in understanding cancer evolution and target therapy. Molecular photography of a tumor allows us to establish which cellular mechanisms are altered and draw specific therapies directly to these molecular targets, decreasing the side effects on healthy cells. From a “one size fits all medicine” to a personalized and specific point of view where therapy is established on the molecular profile of the single tumor in an individual patient, the main objectives in cancer medicine are to maximize the care potential, minimize the toxicity, and identify the patients who will be able to benefit from the therapy [3,4,5,6]. The molecular analysis requires a sensitive and accurate estimate of cancer risk conferred by genetic alterations [7]. 

The mutated genes most often requested by clinicians to choose the appropriate targeted therapy are those of patients affected by NSCLC, mCRC, and melanoma. The prognosis of lung cancer is currently very low; many patients, more than 50%, die within the first year of diagnosis, and after 5 years the survival rate is about 18% [8]. Lung cancer is divided into two major subtypes: small-cell lung carcinoma (SCLC) and non-small-cell lung cancer (NSCLC), accounting for 15% and 85% of all lung cancer, respectively [9]. The most common type of lung cancer is adenocarcinoma in both adults and in younger patients [10]. 

In recent years, the use of target therapy and immunotherapy have conducted a positive management of lung cancer patients [9,11]. The search for tumor mutational burden (TMB) predictive to response to therapy contributed to an increased rate of disease-free survival in patients affected by NSCLC, with respect to those treated by standard chemotherapy [12]. Personalized medicine responded conspicuously in all those patients who had mutations of the EGFR or ALK gene [13]. There are many alterations that are being sought today and that would be targeted as therapy, such as the rearrangement of ROS1 and RET, the amplification of MET, and activating mutations in BRAF, HER2, and KRAS genes [11]. Some genetic variants of colorectal cancer are much rarer and require treatment paths that are different from the first-line chemotherapies generally used. More aggressive and rapidly progressive colorectal cancers are frequently associated with a mutation in the KRAS gene, which encodes an EGFR-activated protein. Genetic tests in a population of mCRC patients have identified that the KRAS gene mutation is common to 40% of cases and it was associated with a failure to respond to standard treatment. Identifying the molecular bases that characterize the tumor is important to define a personalized therapeutic plan: the presence of some genetic mutations, in fact, is predictive of a specific therapy efficacy and allows practitioners to choose the most appropriate drugs for the patient [14,15].

About 90% of the mutations occur at the level of the MSH2 and MLH1 genes (60% in MSH2 and 30% in MLH1), while the PMS1 and PMS2 genes are rarely mutated. When a mutational event occurs at the level of one of these genes, the DNA mismatch repair decreases, and consequently the cell begins to accumulate multiple somatic mutations and develops cancer [16]. These errors occur mainly in repetitive DNA fragments (microsatellites) scattered throughout the genome, resulting in mutations in various target genes. Finally, a third route of carcinogenesis recently identified in the field of epigenetics, as a gene silencing mechanism, is the aberrant hypermethylation of tumor suppressor genes [17]. 

Melanoma is a skin tumor developed by the degeneration of melanocytes following a complex interaction between exogenous and endogenous triggers, as well as tumor-intrinsic and immune-related factors. Similarly to other cancers, malignant transformation into melanoma conforms to a sequential genetic model and subsequent activation of oncogenic signal transduction. Melanocyte proliferation is attributed to the oncogenic mutation of the BRAF gene and, above all, to the rearrangement of chromosome 9p21. In fact, this structural chromosome abnormality leads to the overexpression of the AKT3 protein, which regulates cell signaling in response to growth factors and is involved in different biological processes, such as cell proliferation, differentiation, apoptosis, and the tumorigenesis).

In high-risk operable or in inoperable stage III and IV melanoma, the most frequently mutated genes are BRAF, NRAS, and c-KIT. The evaluation of mutational status of these genes allow patients to benefit from specific treatment with molecular targeted drugs. The first gene examined is BRAF, given its high frequency of mutations in melanoma (36–52%). In particular, the V600 mutation must be sought, since it represents over 95% of BRAF mutations. If BRAF mutations are not present it is necessary to investigate the mutational status of NRAS gene because mutations of these two genes are mutually exclusive. In BRAF/NRAS, wild-type melanomas and the mutations or amplifications of the c-KIT gene must be analyzed [18,19]. 

A recent study evaluated the efficacy of Ipilimumab as an adjuvant treatment for patients with stage III melanoma who were at a high risk of recurrence. Ipilimumab is an anti-CTLA-4 monoclonal antibody that promotes the activation of T lymphocytes and stimulates the immune response against melanoma [20].

Next generation sequencing (next generation sequencing, NGS) refers to the nucleic acid sequencing technologies united by the ability to sequence, in parallel, millions of DNA fragments.

These technologies have marked a revolutionary turning point for the possibility of characterizing large genomes compared to the first generation of DNA sequencing method (Sanger sequencing), due to the capacity to provide, in a single analysis session, an amount of genetic information millions of times bigger [21,22].

The therascreen RGQ PCR methods are a real-time qualitative PCR assay used on the Rotor-Gene Q MDx instrument for the detection of somatic mutations, using DNA extracted from formalin-fixed paraffin-embedded (FFPE). Using Scorpions and ARMS (Allele Refractory Mutation System) technologies, the therascreen RGQ PCR technologies enable the detection of several mutations in codons of the human oncogenes against a background of wild-type genomic DNA [23,24,25]. 

The purpose of this study was to analyze the usefulness of two different methods, NGS platform Ion Torrent Personal Genome Machine, and Therascreen Rotor Gene Q for the evaluation of the mutational status of target genes. We focused our attention on the examination of mutated genes in NSCLC, mCRC, and melanoma.

## 2. Materials and Methods 

**Sample preparation.** From April 2017 to December 2018 we collected 212 samples from patients enrolled by the university hospital “Luigi Vanvitelli” of Naples: 34 NSCLC, 145 mCRC (Table 1), and 33 melanomas (Table 2). The clinical data of NSCLC patients are not available.

We selected appropriate formalin-fixed paraffin embedded (FFPE) slides for each case. We used four unstained FFPE tissue sections of 10 µm. DNA was obtained using the QIAamp^®^ DNA FFPE kit Tissue (Qiagen, Hilden, Germany), according to the manufacturer’s instructions. Extracted DNA was eluted in 20 or 30 µL of elution buffer and then quantified by Qubit^®^ 2.0 Fluorometer (Life Technologies, Carlsbad, CA, USA) using the Qubit^®^ dsDNA HS Assay kit (Life Technologies, Carlsbad, CA, USA). The DNA samples were stored at −20 °C.

For the massive parallel sequencing of DNA libraries, we used *ION Torrent Personal Genome Machine* (PGM™, Thermo Fisher Scientific, Waltham, MA, USA) that exploits the pH variations occurring during the incorporation of the single deoxyribonucleotide into the reaction catalyzed by the DNA polymerase. Using 10 ng of DNA input, we prepared the sequencing libraries. In detail, the libraries were prepared with IonAmpliSeq™ Library kit 2.0 (Thermo Fisher Scientific, Waltham, MA, USA) and two types of primer pool: IonAmpliSeq Colon and Lung Cancer Research Panel v2, to analyze 504 mutational hotspots and targeted regions in 22 genes (Table 3), and AmpliSeq Cancer Hotspot Panel v2 to scan 2800 mutational hotspots and targeted regions in 50 genes (Table 4). 

Amplified products were purified with Agencourt AMPure XP beads (Beckman Coulter Genomics, High Wycombe, UK). Concentrations of amplified and barcoded libraries were quantified by Qubit^®^ dsDNA HS Assay kit at Qubit ^®^ 2.0 Fluorometer. DNA libraries were stored at −20 °C. The libraries were diluted to 100 pM and clonally amplified on Ion Sphere TM particles. We used the IonOneTouch™ 2 System (Thermo Fisher Scientific, Waltham, MA, USA) to perform emulsion PCR, cover the Ion Sphere™ Particles, and enrich the particles with a positive template. After a short centrifugation step, the Ion Sphere™ particles coated with template were deposited into the wells of the semiconductor chip. Finally, sequencing was carried out using 3.16 (8 samples) or 3.18 (16 samples) chips on the Ion Personal Genome Machine System.

**Data Analysis.** We used the Torrent Suite Software v.4.0.2 (Life Technologies, Carlsbad, CA, USA) to assess run performance and data analysis. Integrative Genomics Viewer (IGV v 2.2, Broad Institute) was used for visual inspection of the aligned reads. The BED files from torrent suite were analyzed using Ion Reporter software [26] and filtered according to the quality control (specified by the panel datasheet as indicated by manufacturer instructions, Table 3 and Table 4). We selected the SNVs resulting in a non-synonymous amino acid change, or a premature stop codon, and in short indels resulting in either a frame-shift or insertion/deletion of amino acids. All SNVs were analyzed for previously reported hotspot mutations (somatic mutations described in COSMIC database) and novel variations, i.e., new mutations detected by NGS but not reported in either COSMIC or db SNP databases. 

**Therascreen Rotor Gene Q.** Rotor-Gene Q MDx instrument was used to perform a real-time qualitative PCR assay for the detection of somatic mutations in the human oncogene against a background of wild-type genomic DNA, using DNA extracted from formalin-fixed paraffin-embedded (FFPE) samples. This real-time PCR combines an amplification refractory mutation system (ARMS) and a Scorpion fluorescent primer/probe system. 

Allele-specific amplification is achieved by ARMS, which exploits the ability of Taq DNA polymerase to distinguish between a matched and a mismatched base at the 3’ end of a PCR primer. When the primer is fully matched, the amplification proceeds with full efficiency. When the 3’ base is mismatched, only low-level background amplification may occur. Therefore, a mutated sequence is selectively amplified even in samples where the majority of the DNA does not carry the mutation.

Detection of amplification is performed using Scorpions. Scorpions are bifunctional molecules containing a PCR primer covalently linked to a probe. The probe incorporates the fluorophore carboxyfluorescein (FAM™) and a quencher. The latter quenches the fluorescence of the fluorophore. When the probe binds to the ARMS amplicon during PCR, the fluorophore and quencher become separated, leading to a detectable increase in fluorescence [23,24,25]. Therascreen EGFR RGQ PCR kit enables the detection of 29 mutations in exons 18, 19, 20, and 21 of EGFR oncogene (19 deletions in exon 19; 3 insertion in exon 20; G719X, that detects the presence of G719S, G719A, or G719C but does not distinguish between them; S768I, T790M, L858R, and L861Q). Therascreen KRAS RGQ PCR kit enables the detection of 7 mutations in codons 12 and 13 of the KRAS oncogene (G12A, G12D, G12R, G12C, G12S, G12V, G13D). Therascreen BRAF RGQ PCR kit enables the detection of the following mutations: V600E/V600E complex, V600D, V600K, and V600R at codon 600 of exon 15 of the BRAF oncogene.

## 3. Results

We analyzed 212 samples of patients enrolled by the university hospital “Luigi Vanvitelli” of Naples: 34 NSCLC, 145 mCRC, and 33 melanomas. After DNA extraction, samples were analyzed with Ion Torrent Personal Genome machine, and coding and amino acid change data of mutated genes available in the panel have been exported in a database. We focused our attention on the EGFR gene for NSCLC samples, KRAS, and BRAF genes for mCRC samples, and the BRAF gene for melanoma samples, because target therapy for the mutation of these genes can be prescribed by clinicians. 

Extracted DNA was then subjected to further analysis by Therascreen PCR for the mutations detectable by the following kits: Therascreen EGFR RGQ PCR kit for NSCLC samples, Therascreen KRAS RGQ PCR kit, Therascreen BRAF RGQ PCR kit for mCRC samples, and Therascreen BRAF RGQ PCR kit for melanoma samples.

The results about target oncogene status were then compared. 

Our study focused on three different directions: NGS analysis versus Therascreen KRAS RGQ PCR, NGS analysis versus Therascreen BRAF RGQ PCR, and NGS analysis versus Therascreen EGFR RGQ PCR analysis. 

In 145 mCRC samples, we found comparable results for KRAS gene status for the mutations detectable by KRAS RGQ PCR kits. In detail, we found 109 wild-type and 36 mutated samples: the mutation most frequently found involved the substitution of glycine with asparagine in codon 12 of exon 2 of KRAS gene (13 samples 8.96% of frequency) (Table 5).

Extracted DNA from 145 mCRC samples was then subjected to analysis with BRAF RGQ PCR kits. In this case we did not find comparable results for 2 out of 145 samples. In the first case, BRAF gene mutation profile by qualitative real-time PCR showed the presence of p.Val600Arg (c.1798_1799delGTinsAG) mutation on exon 15, not detected by NGS platform. 

In the second case, p.Val600Glu (c.1799T>A) mutation on exon 15 exon BRAF was identified by qualitative real-time PCR gene analysis which showed its presence, but it was not detected by the NGS assay (Table 6). This mutation results in an amino acid substitution at position 600 in BRAF, of valine (V) for glutamic acid (E) and subsequent increased kinase activity. 

We found comparable results for BRAF gene status in melanoma samples by two types of methodologies (Table 7). 

In 32 out of 34 NSCLC samples, the results were equivalent. EGFR (epidermal growth factor receptor, also known as ERBB1 and HER1) is a gene that encodes for the EGFR protein: activating EGFR mutations increase the kinase activity of EGFR, leading to hyperactivation of downstream pro-survival signaling pathways. EGFR is mutated in 22.2% of NSCLC patients, with L858R EGFR mutation present in 6.05% of all NSCLC patients. In one patient, EGFR gene analysis by qualitative real-time PCR showed the presence of a p.Leu858Arg (c.2573T>G) mutation on exon 21. This mutation was not detected by the NGS platform. Leu858Arg mutation results in an amino acid substitution at position 858 in EGFR, from a leucine (L) to an arginine (R). This mutation on exon 21 occurs in approximately 43% of EGFR-mutated lung tumors and affects the kinase domain.

In the second case, DNA analysis by Therascreen EGFR RGQ PCR showed the presence of the classical deletion on exon 19 that confers increased sensitivity to EGFR Tyrosine Kinase Inhibitors (TKIs) (Table 8).

In the second case, the first analysis by Ion Torrent Personal Genome Machine did not detect the presence of deletion on exon 19 of the EGFR gene but showed a non-canonical mutation on the same exon (19) of EGFR gene (p.Leu747Ser c.2240T>C).

## 4. Discussion 

In this study we evaluated the usefulness of two methods, NGS platform Ion Torrent Personal Genome Machine and Therascreen Rotor Gene Q [27], for the detection of target gene mutations in NSCLC, mCRC, and melanoma patients, mutations for which an alternative clinical therapy to chemotherapy is envisaged. 

For the identification of mutation profile in cancer patients, it is necessary to use alternative methods of analysis with different percentages of accuracy and sensitivity to detect a mutation-driven drug resistance. Ion Torrent Personal Genome Machine has a sensitivity of results of 95% [28], while the qualitative real time PCR Rotor gene Q has a sensitivity of 99%. In our cohort of patients, the use of the two methods allowed us to identify mutations that otherwise we would have not found, with negative consequences on the clinical choices for the patients. In fact, clinical guidelines (AIOM—Italian Association of Medical Oncology) for treatment of cancer patients suggest a different therapy based on mutation detected by molecular analysis. 

KRAS mutation is an established predictive biomarker for anti-EGFR therapy resistance. The KRAS oncogene produces colorectal tumors resistant to anti-EGFR therapies by activating the Ras-Raf-MAPK pathway downstream of EGFR. 

KRAS is mutated in 47.0% of mCRC patients, with KRAS p.Gly12Asp recorded in 13.3% of all mCRC patients; this activation mutation is the most frequent KRAS mutation in mCRC patients [29]. In our cohort, we found 109 wildtype and 36 mutated samples. According to the literature, the mutation most frequently found involves the substitution of glycine with asparagine in codon 12 of exon 2 of KRAS gene (13 samples, 8.96% of frequency).

Mutation of the BRAF proto-oncogene is linked to a variety of cancers and it is used as a prognostic tool and therapeutic target. Oncogenic mutations in BRAF are present in 10% of mCRC, and BRAF status is believed to be responsible for the 12–15% of patients who fail to respond to anti-EGFR therapy [30]. The role of BRAF mutation status as a predictive molecular marker is less clear. The most investigated and predictive role of BRAF mutation is like a biomarker of anti-EGFR antibody resistance.

Several studies have suggested that BRAF mutations also confer poor outcomes with anti-EGFR therapy [30]. Of 145 mCRC samples analyzed with BRAF RGQ PCR kits, we did not find comparable results for two of them. In the first case, the BRAF gene mutation profile by qualitative real-time PCR showed the presence of p.Val600Arg (c.1798_1799delGTinsAG) mutation on exon 15. This mutation was not detected by the NGS platform. It results in amino acid substitution at position 600 in BRAF, of valine (V) for arginine (R), and occurs within the activation segment of the kinase domain, resulting in increased kinase activity. Clinical data about this patient were in accordance with the results obtained by therascreen analysis. In fact, a first-line chemotherapy treatment according to the Folfox scheme with the addition of bevacizumab, in agreement with the RAS WT and BRAF mut V600R status, was started. In view of the pulmonary progression disease, the patient received 2nd line treatment according to the Folfiri + Aflibercept scheme, performing 18 total cycles. In III line, regorafenib administration was started but the treatment was suspended for new pulmonary progression diseases. As expected, resistance to regorafenib was predictable for the presence of BRAF mutation in the tumor. In the second case, BRAF gene analysis by qualitative real-time PCR showed the presence of p.Val600Glu (c.1799T>A) mutation on exon 15 exon but it was not detected by the NGS assay in this sample. It results in an amino acid substitution at position 600 in BRAF, of valine (V) for glutamic acid (E), and subsequent increased kinase activity. Activating hotspot V600E mutation contributes to constitutive activation of MAPK signaling and uncontrolled cellular growth. For mCRC samples, the presence of BRAF V600E mutation may be indicative of resistance to treatment with anti-EGFR monoclonal antibodies and this information helps the clinicians to have more stringent monitoring of the mutated BRAF patient than wildtype, because the resistance to anti-EGFR therapy could occur more rapidly [31].

We found comparable results for BRAF gene status in melanoma samples by two types of methodologies.

EGFR (epidermal growth factor receptor, also known as ERBB1 and HER1) is a gene that encodes for the EGFR protein: activating EGFR mutations increase the kinase activity of EGFR, leading to hyperactivation of downstream pro-survival signaling pathways. EGFR is mutated in 22.2% of NSCLC patients, with the L858R EGFR mutation present in 6.05% of all NSCLC patients. 

For NSCLC patients, using two methods, we were able to identify the presence of EGFR mutations in two patients, which were identified as wild-type at the Ion Torrent Personal Genome machine analysis. In detail, we found EGFR Exon 19 Deletion in one patient and the EGFR-L858R mutation of EGFR in another patient. In both cases, the patients can be treated with TKIs to give them an additional chance of treatment with a consequently higher expectancy of life [32,33]. This is still more relevant in the era of the second and third generation TKIs and requires a more precise and deep molecular characterization of NSCLC. 

We hypothesize that the small cellular clone not detectable by NGS was indeed mutated, but the major part of the cancer cell population did not express the mutation. Therefore, it is becoming relevant to use an additional method with increased sensitivity and accuracy for the study of patients who were confirmed as wildtype at the NGS platform. It is easy to detect a mutation, but diagnosing a wildtype sample can be complicated. In our current practice, we routinely use both methods for the patients who were confirmed as wildtype at NGS in order to confirm the results or to research the small clone that, in the future, can response to the target therapy. 

## 5. Conclusions

In summary, we compared two methods for the detection of somatic cancer variants in FFPE specimens. Therascreen assay is appropriate for use in routine clinical testing as a result of having more specific and sensitive mutation detection than Ion Torrent PGM. 

**Figure 1:**
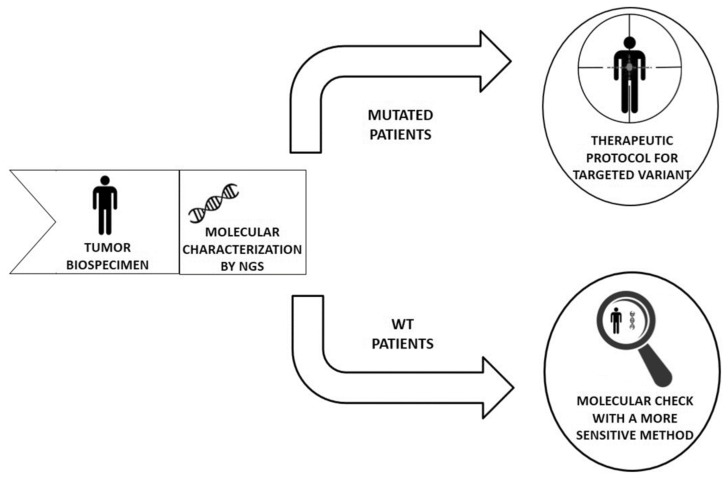
Diagnosis of the wild-type status in cancer patients requires more sensitive methods in order to identify a small mutated and neoplastic clone otherwise not detectable with NGS techniques.

These methods are sample-saving, cost-efficient, and a time-efficient platform for multiplex genetic testing in different cancers with great potential for clinical application, as it is conceivable to include new mutations to test in other genes. In detail, for NGS molecular analysis by the PGM method we need 1 week, while for qualitative PCR analysis we need 2 days; the shorter time needed to give the result allows to the clinician to rapidly administrate the target therapy for cancer patients. At the moment it is very difficult to determine the wild-type status of genes on tissue samples (Figure 1). Therefore, we suggest adopting a more sensitive method to clearly confirm the absence of mutations in genes classified as wild-type with massively parallel sequencing. Considering that these patients have a small neoplastic clone harboring the mutation, it is conceivable that after chemotherapy the mutated neoplastic clone takes over and the patient become sensitive to the specific drug therapy. 

## Figures and Tables

**Table 1:** Clinical data of 145 colon cancer patients.

Characteristics	Number of Patients (%)
**Age (years)**	
Mean	64.95
Median	66
**Gender**	
Male	85 (*58.3%*)
Female	60 (*41.7%*)
**Tumor localization**	
left colon	48 (*33.3%*)
left colon	15 (*10.4%*)
right colon	41 (*28.5%*)
sigmoid	10 (*6.9%*)
rectal	29 (*20.1%*)
right and left colon	2 (*0.8%*)
**Tumor grading**	
G1	10 (*7.2%*)
G2	86 (*59.3%*)
G2 mucinous	8 (*5.3%*)
G3	41 (*28.2%*)

**Table 2:** Clinical data of 33 melanoma cancer patients.

Characteristics	Number of Patients (%)
**Age (years)**	
Mean	58.78
Median	59.39
**Gender**	
Male	21 (*63%*)
Female	12 (37%)
**Primary tumor site**	
head and neck	4 (*12.2%*)
trunk	18 (*54.5%*)
extremities (E)	11 (*33.3%*)
**Tumor depth (Breslow thickness)**	
**Average depth**	
<1.0 mm	1 (*3.0%*)
1.01–2.0 mm	1 (*3.0%*)
2.01–4.0 mm	4 (*12.1%*)
>4.01 mm	27 (*81.9%*)
**SLN (sentinel lymph node)**	
YES	17 (*51.5%*)
NO	16 (*48.5%*)
**TNM**	
pT2	1 (*3.0%*)
pT3a	4 (*12.1%*)
pT3b	2 (*6.0%*)
pT4a	5 (*15.1%*)
pT4b sec.AJCC VIII ed.	21 (*63.8%*)
**STAGE**	
IIIA	1 (*3.0%*)
IIIB	0 (*0%*)
IIIC	6 (*18.2%*)
IV	26 (*78.8%*)

**Table 3:** IonAmpliSeq Colon and Lung Cancer Research Panel v2.

Sample Type	FFPE Samples
**APPLICATION**	Somatic mutation detection
**GENES**	**KRAS, EGFR, BRAF, PIK3CA, AKT1,ERBB2, PTEN, NRAS, STK11, MAP2K1, ALK, DDR2, CTNNB1, MET, TP53, SMAD4, FBX7, FGFR3, NOTCH1, ERBB4, FGFR1, FGFR2**
**PRIMER PAIRS, AMPLICON LENGHT**	92 pairs of primers in a single pool 92 amplicons with an average length of 162 bp
**IMPUT DNA REQUIRED**	10 ng
**OBSERVED PERFORMACE**	Percent of amplicons with the target base coverage at 500x: >95% Average panel uniformity: 95% Average percent reads on target: 98%

**Table 4:** AmpliSeq Cancer Hotspot Panel v2.

Sample Type	FFPE Samples
**APPLICATION**	Somatic mutation detection
**GENES**	**ABL1, AKY1, ALK, APC, ATM, BRAF, CDH1,CDKN2A, CSF1R, CTNNB1,EGFR, ERBB2, ERBB4, EZH2,FBWX7, FGFR1, FGFR2, FGFR3, FLT3, GNA11, GNAS, GNAQ, HNF1A, HRAS, IDH1, JAK2, JAK3, IDH2, KDR, KIT, KRAS, MET, MLH1, MPL, NOTCH1, NPM1, NRAS, PDGFRA, PIK3CA, PTEN, PTPN11, RB1, RET, SMAD4, SMARCB1, SMO, SRC, STK11, TP53, VHL**
**PRIMER PAIRS, AMPLICON LENGHT**	207 pairs of primers in a single pool 111–187 bp, average length of 162 bp
**IMPUT DNA REQUIRED**	10 ng
**OBSERVED PERFORMACE**	Percent of amplicons with the target base coverage at 1400x: >95% Average panel uniformity: 95% Average percent reads on target: 98%

**Table 5:** Percentage of KRAS mutations detected in mCRC patients by using Ion Torrent Personal Genome Machine and Therascreen KRAS RGQ PCR kit.

KRAS	PGM KRAS	Thera KRAS
WT	109 (75.7%)	109 (75.7%)
Mut RAS		
Gly12Ala	3 (2.1%)	3 (2.1%)
Gly12Asp	13 (8.9%)	13 (8.9%)
Gly12Arg	0	0
Gly12Cys	2 (1.4%)	2 (1.4%)
Gly12Ser	1(0.7%)	1(0.7%)
Gly12Val	6 (4.1%)	6 (4.1%)
Gly13Asp	11 (7.6%)	11 (7.6%)

**Table 6:** Percentage of BRAF mutations detected in mCRC patients by using Ion Torrent Personal Genome Machine and Therascreen BRAF RGQ PCR kit.

BRAF Status	PGM BRAF	Thera BRAF
WT BRAF	135 (*93.1*%)	133 (*75.7*%)
Mut BRAF		
V600E	10 (*6.9*%)	11 (*7.6*%)
V600D	0	0
V600K	0	0
V600R	*0*	1 (*0.7*%)

**Table 7:** Percentage of BRAF mutations detected in melanoma patients by using Ion Torrent Personal Genome Machine and BRAF RGQ PCR kits.

BRAF Status	PGM BRAF	Thera BRAF
WT BRAF	16 (48.5%)	16 (48.5%)
Mut BRAF		
V600E	12 (36.4%)	12 (36.4%)
V600D	0	0
V600K	5 (15.1%)	5 (15.1%)
V600R	0	0

**Table 8:** Percentage of EGFR mutations detected in NSCLC patients by using Ion Torrent Personal Genome Machine and Therascreen EGFR RGQ PCR kit.

EGFR Status	PGM EGFR	Thera EGFR
WT EGFR	29 (*85.2%*)	27 (*79.4%*)
Mut EGFR		
T790M	1 (2.9%)	1 (2.9%)
Del	4 (*11.7%*)	5 (*14.7%*)
L858R	0	1 (2.9%)
L861Q	0	0
G719X	0	0
S768I	0	0
Ins	0	0
